# Dimethylarginines in pediatric CKD: clinical utility of ADMA and SDMA as biomarkers

**DOI:** 10.3389/fped.2025.1662259

**Published:** 2025-11-12

**Authors:** Hülya Nalçacıoğlu, Murat Cihan, Hülya Gözde Önal, Demet Tekcan Karalı

**Affiliations:** 1 Department of Pediatric Nephrology, Medical Faculty, Ondokuz Mayis University, Samsun, Türkiye; 2 Department of Ordu University Training and Research Hospital, Department of Biochemistry, Ordu, Türkiye

**Keywords:** chronic kidney disease, pediatrics, biomarkers, asymmetric dimethylarginine, symmetric dimethylarginine

## Abstract

**Introduction:**

Pediatric chronic kidney disease (CKD) often presents no symptoms in its early stages, making timely diagnosis challenging. Asymmetric dimethylarginine (ADMA) and symmetric dimethylarginine (SDMA) are methylated arginine derivatives that reduce nitric oxide availability and have been suggested as potential early indicators of kidney dysfunction. This study aimed to evaluate the usefulness of ADMA and SDMA in pediatric CKD and to assess their association with renal function and disease severity.

**Methods:**

This single-center, cross-sectional, observational study enrolled 100 children aged 1–18 with CKD stages 2–4 and 70 healthy, age- and sex-matched controls between January and September 2023. Serum ADMA and SDMA levels were measured using ultra-high-performance liquid chromatography-tandem mass spectrometry (UHPLC-ESI-MS/MS). The estimated glomerular filtration rate (eGFR) was calculated using the Schwartz formula. Comparisons were made between groups and CKD stages, and correlation analyses were conducted with eGFR.

**Results:**

ADMA and SDMA levels were significantly higher in the CKD group than in the control group (*p* < 0.001 and *p* = 0.013, respectively), while the ADMA/SDMA ratio showed no significant difference. ADMA levels increased progressively with CKD stage, particularly in stage 4 patients (*p* < 0.001). There were moderate negative correlations between eGFR and both ADMA (r = −0.380, *p* < 0.001) and SDMA (r = −0.238, *p* = 0.002). These findings suggest that both biomarkers increase with disease progression, with ADMA demonstrating moderate associations.

**Conclusion:**

Serum ADMA and SDMA levels increase with worsening kidney function in children and may serve as useful markers associated with disease severity in pediatric CKD, but further validation is required. In particular, ADMA reflects disease severity and endothelial dysfunction, highlighting its potential role in clinical risk stratification.

## Introduction

Chronic kidney disease (CKD) is characterized by a glomerular filtration rate (GFR) below 60 ml/min/1.73 m^2^ persisting for ≥3 months or the presence of structural or functional kidney damage markers (1). The leading causes of pediatric CKD include congenital anomalies, obstructive uropathies, and hereditary nephropathies, which often present asymptomatically during early stages (2). As CKD progresses from stages 2 to 4, metabolic complications such as acidosis, anemia, growth retardation, and secondary hyperparathyroidism emerge and intensify parallel to declining GFR (3). Therefore, identifying CKD early through biochemical markers is essential for slowing disease progression and enhancing patient quality of life.

Recent research has indicated methylated arginine derivatives, including asymmetric dimethylarginine (ADMA) and symmetric dimethylarginine (SDMA), as potential biomarkers for CKD due to their impact on endothelial function and nitric oxide synthesis (NOS). ADMA, an endogenous inhibitor of NO synthase, contributes to endothelial dysfunction. In contrast, SDMA indirectly decreases NOS availability by inhibiting L-arginine transport despite not directly inhibiting NOS (4, 5). Since the kidneys clear both molecules, their serum concentrations significantly rise in patients with impaired kidney function. Additionally, increased ADMA levels correlate strongly with elevated cardiovascular risks and mortality among adults (6).

While limited, pediatric studies examining ADMA and SDMA in relation to renal function have yielded significant insights (7). Wasilewska et al. demonstrated markedly elevated SDMA even in early pediatric CKD stages, suggesting its sensitivity as a marker for early renal dysfunction (7). Similarly, Piechowicz et al. reported significant variations in ADMA, SDMA, and citrulline levels by CKD stage in children, with ADMA negatively correlated with eGFR (5). However, some studies note that the ADMA/SDMA ratio alone may not significantly reflect CKD progression (8).

This study aimed to assess serum ADMA and SDMA levels in pediatric patients with CKD stages 2–4 and explore their associations with disease severity and renal function. Additionally, we aimed to evaluate the potential of these biomarkers for early diagnosis and prognosis of CKD by comparing them with healthy pediatric controls. This research contributes to the limited evidence on the clinical utility of ADMA and SDMA in pediatric nephrology.

## Materials and methods

### Study design and participants

This study was designed as a single-center, cross-sectional observational investigation. It included 100 pediatric patients diagnosed with CKD attending the Pediatric Nephrology outpatient Clinic of Ondokuz Mayıs University between January 2023 and September 2023, along with 70 age- and sex-matched healthy controls. CKD diagnosis was established based on the Kidney Disease Improving Global Outcomes (KDIGO) criteria, defined as eGFR <90 ml/min/1.73 m^2^ and/or structural/functional markers indicative of kidney damage (1).

### Inclusion and exclusion criteria

The inclusion criteria for patients were: (1) age between 1 and 18 years, (2) CKD diagnosis established at least 3 months prior, and (3) being classified within CKD stages 2, 3, or 4. The control group comprised healthy children without any previous kidney disease or systemic illness history. Exclusion criteria included diabetes, acute infections, malignancy, liver disease, congenital metabolic disorders, active inflammatory conditions, and ongoing steroid or immunosuppressive therapy.

### Ethical approval

The study protocol received approval from the Clinical Research Ethics Committee of Ordu University, and informed written consent was obtained from the parents of all participating children (KAEK 164, 2022). The study was conducted following the Declaration of Helsinki

### Data collection

Demographic and clinical data, including age, sex, height, weight, body mass index (BMI), CKD etiology, CKD stage, and number of antihypertensive medications, were recorded. Renal function was assessed by calculating eGFR using the Schwartz formula. Laboratory parameters measured included serum creatinine, ADMA, and SDMA.

### Measurement of ADMA and SDMA

Blood samples were centrifuged within 30 min of collection. Serum aliquots were frozen at −80°C and stored for up to 6 months before analysis. Samples with visible hemolysis, lipemia, or icterus were excluded. All measurements were performed in a single analytical batch to minimize inter-assay variability. Serum ADMA and SDMA levels were quantified using ultra-high performance liquid chromatography-electrospray ionization tandem mass spectrometry (UHPLC-ESI-MS/MS), following the method described by Di Gangi et al. (9). The analysis utilized mobile phase A, containing 40 mM ammonium formate and 3% formic acid, and mobile phase B consisting of acetonitrile. Samples were analyzed under isocratic and gradient flow conditions using a Phenomenex polar RP column (75 × 4.6 mm × 4 µm). The instrument settings were calibrated according to precursor and product ion m/*z*-values and collision energies as specified by Di Gangi et al. (9).

### Statistical analysis

Data analysis was performed using IBM SPSS Statistics version 26.0 (IBM Corp., Armonk, NY, USA). Continuous variables were assessed for normality using the Shapiro–Wilk test, and variables that did not show normal distribution were reported as median (IQR). The Mann–Whitney *U*-test was used for comparisons between groups, while the Kruskal–Wallis test was applied for comparisons among CKD stages. Correlation analyses were conducted using Spearman's correlation coefficient. A *p*-value of less than 0.05 was considered statistically significant.

## Results

In this study, data from a total of 100 pediatric patients diagnosed with CKD stages 2–4 and 70 age- and sex-matched healthy controls were analyzed. The participants' demographic characteristics, clinical findings, and biomarker levels were compared.

Table 1 shows no statistically significant differences between the CKD and control groups regarding age, sex, and BMI. In contrast, eGFR levels were significantly lower in the CKD group (*p* < 0.001). As indicated in Table 2, ADMA and SDMA levels were significantly higher in the CKD group compared to the control group (*p* < 0.001 and *p* = 0.013, respectively). However, no significant difference was observed between the groups regarding the ADMA/SDMA ratio (*p* = 0.392).

**Table 1:** Demographic data of the studied subjects.

Data	Variables	Patients with CKD	Controls	*p*-value
Number		100	70	
Age(years), median (min-max)		8.5 (1–17)	10 (2–17)	0.064
Gender,female,number		35	35	0.058
BMI (kg/m^2^)		19.5 (12.35–39.86)	18.02 (11.48–32)	0.06
Etiology of CKD *n* (%)	CAKUT	64		
	Neurogenic bladder	15		
	Polycystic kidney disease	4		
	Glomerulonephritis- HUS	7		
	Undetermined	10		
CKD stage, *n* (%)	CKD stage 2	47		
	CKD Stage 3	34		
	CKD Stage 4	19		
eGFR median, IQR (mL/min/1.73 m^2^)		61.58 (43.31)	112,38 (19.94)	<0.001
Number of antihypertensive agents, number	None	68		
	+1	27		
	+2	3		
	+3	2		

CAKUT, congenital anomalies of the kidney and urinary tract; HUS, hemolytic uremic syndrome; eGFR, estimated glomerular filtration rate (mL/min/1.73 m^2^).

**Table 2:** Comparison of plasma ADMA and SDMA levels between the CKD and control subjects.

Variable	CKD patients	Control	*p*-value
ADMA (nmol/L), median (IQR)	83 (90)	51 (40)	<0.001
SDMA (nmol/L), median (IQR)	225 (470)	184 (110)	0.013
ADMA/SDMA ratio	0.266 (0.11)	0.254 (0.09)	0.392

Values are expressed as median (IQR). Units are nmol/L unless otherwise specified.

ADMA, asymmetric dimethylarginine; SDMA, symmetric dimethylarginine; CKD, chronic kidney disease; IQR, interquartile range.

**Figure 1:**
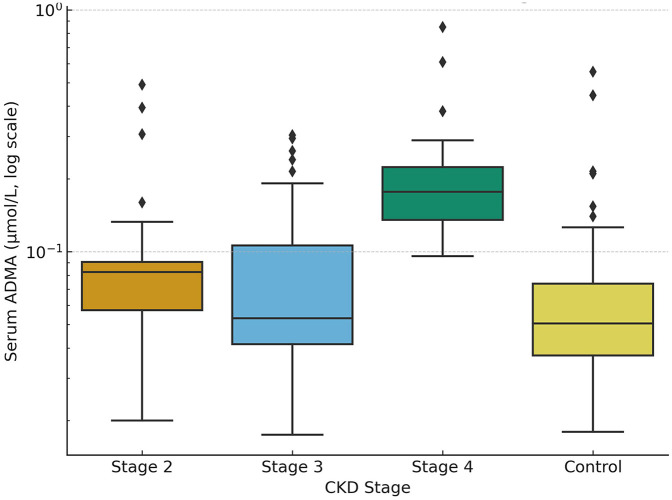
Serum ADMA levels (µmol/L) across pediatric CKD stages (stage 2, stage 3, stage 4) and healthy controls. Box plots represent the median, interquartile range (IQR), and outliers, with individual values overlaid as scatter points. The *y*-axis is displayed on a logarithmic scale to better illustrate inter-group differences and the wide distribution of higher ADMA values.

When evaluated by CKD stages, ADMA levels demonstrated a significant increase with disease progression. Children in stage 4 CKD had significantly higher ADMA levels compared to both the control group and those in stages 2 and 3 (*p* < 0.001). Although ADMA levels in stage 3 were higher than those in the control and stage 2 groups (*p* = 0.035), the difference between stages 2 and 3 was not statistically significant (*p* = 0.368). According to *post-hoc*, Dunn–Bonferroni corrected analysis results, no significant difference was detected between the control and stage 2 groups (*p* = 1.000). In contrast, significant differences were observed between the control group and both stage 3 (*p* = 0.035) and stage 4 groups (*p* < 0.001). Additionally, significant differences were noted between stage 2 and stage 4 (*p* < 0.001) and between stage 3 and stage 4 (*p* = 0.003) (Figure 1, Table 3). These findings support that ADMA levels increase markedly, particularly in advanced stages, and may serve as a potential biomarker reflecting disease progression in pediatric CKD.In addition, absolute differences with 95% confidence intervals were calculated. For ADMA, CKD patients had a higher median value compared to controls (median difference: 0.061 µmol/L, 95% CI: 0.044–0.079, *p* < 0.001). For SDMA, the difference was smaller but still significant (median difference: 0.018 µmol/L, 95% CI: 0.011–0.025, *p* = 0.002).

**Table 3:** Pairwise comparisons of ADMA between groups.

Comparison	Median difference (µmol/L)	95% CI	*p* (Bonferroni)
Stage 2 vs. Control	0.032	0.008–0.039	0.012
Stage 3 vs. Control	0.003	−0.010–0.012	1.000
Stage 4 vs. Control	0.127	0.082–0.148	<0.0001
Stage 2 vs. Stage 3	0.029	0.006–0.039	0.243
Stage 2 vs. Stage 4	−0.095	−0.132–−0.054	<0.0001
Stage 3 vs. Stage 4	−0.124	−0.149–-0.081	<0.0001

Median differences with 95% confidence intervals (CI) were calculated using bootstrap resampling. *p*-values were adjusted with the Bonferroni method.

ADMA, asymmetric dimethylarginine.

Correlation analysis revealed a moderate negative correlation between ADMA and eGFR (r = –0.380, *p* < 0.001). Similarly, a significant negative correlation was found between SDMA and eGFR (r = –0.238, *p* = 0.002). These results indicate that both biomarkers increase as renal function deteriorates. Overall, ADMA and SDMA levels were observed to rise in parallel with CKD progression, with ADMA exhibiting a more pronounced elevation, particularly in stages 3 and 4, suggesting its potential as a marker of disease progression.

## Discussion

This study aimed to evaluate ADMA and SDMA levels in pediatric CKD patients and investigate the relationship of these biomarkers with disease stages. Our results demonstrated that ADMA and SDMA levels were significantly higher in the CKD group than in healthy controls, and this elevation became more pronounced with increasing disease stages. These findings suggest that ADMA and SDMA may be potential biomarkers for the diagnosis and progression of childhood CKD.

ADMA and SDMA are recognized as endogenous inhibitors of NOS, playing roles in various physiological processes contributing to endothelial dysfunction (4). High ADMA levels in adult CKD patients are associated with accelerated disease progression and increased cardiovascular risk (6). SDMA, the biologically inactive isomer of ADMA, better reflects renal function since it is completely excreted via the renal pathway (10). Studies in adults have reported that SDMA levels closely correlate with declining kidney function, whereas ADMA has stronger associations with hypertension and cardiovascular mortality (10).

Pediatric studies have highlighted the significance of ADMA and SDMA in the context of chronic kidney disease. Wasilewska et al. (7) showed that SDMA increases early in pediatric CKD, correlating strongly with declining eGFR, making it a more sensitive marker of early renal impairment than creatinine. Snauwaert et al. (10) reported that SDMA is elevated across all stages of CKD in children, while ADMA rises only in advanced stages, further emphasizing SDMA's sensitivity. El-Sadek et al. (11) identified SDMA as an independent predictor of reduced eGFR, supporting its clinical utility.

The decline in glomerular filtration rate reduces the renal clearance of dimethylarginines, resulting in their accumulation in plasma (7). Thus, the relationship between declining eGFR and increasing ADMA and SDMA levels forms the foundation of our study. ADMA levels were significantly higher in the CKD group than in the controls. Similarly, Piechowicz et al. (5) reported elevated ADMA and SDMA levels in pediatric CKD cases. El-Sadek et al. (11) also found higher serum ADMA levels in children with CKD compared to healthy controls and those at earlier disease stages. This suggests that ADMA may increase in parallel with disease progression. Furthermore, this study emphasized that SDMA levels were significantly elevated and could be a sensitive marker of renal dysfunction.

Combining ADMA and SDMA measurements may improve clinical accuracy. Benito et al. (12) demonstrated that this combined approach enhances early CKD detection, offering accuracy comparable to creatinine. However, the ability of these markers to predict CKD progression is less clear. While Brooks et al. (13) found that initial ADMA and SDMA levels correlate with renal function but do not reliably predict GFR decline, El-Sadek et al. (11) reported that SDMA might effectively predict worsening renal function. Further studies are needed to confirm these findings.

Consistent with previous studies, Jezierska and Stefanowicz (8) emphasized the moderate negative correlation between SDMA and eGFR and its potential association with the development of hypertension. Additionally, a recent publication in Nature Scientific Reports (14) indicated that SDMA, in combination with creatinine and citrulline, demonstrated high specificity and sensitivity for diagnosing early-stage CKD.

Our findings regarding the ADMA/SDMA ratio are also consistent with previous literature. Jezierska and Stefanowicz (8) noted that evaluating ADMA and SDMA levels separately may be more effective in predicting disease progression, whereas the ADMA/SDMA ratio often does not show significant differences. In our study, no significant difference was observed in the ADMA/SDMA ratio between groups (*p* = 0.392), suggesting that absolute levels are more informative than ratios. Significant negative correlations were observed between both ADMA and SDMA levels and eGFR. Similar results have been reported by Piechowicz et al. (5) and Wasilewska et al. (7), indicating that while ADMA is clinically relevant for endothelial dysfunction, SDMA is a sensitive marker for renal clearance impairment.

The comparison of ADMA levels across CKD stages, as shown in Figure 1 and Table 3, clearly demonstrates the relationship between disease progression and biomarker elevation. Children in stage 4 CKD had significantly higher ADMA levels compared to those in stages 2 and 3 (*p* < 0.001). Similar findings have been reported in studies on adult patients by Guo et al. (15). ADMA has been associated with renal function decline, endothelial dysfunction, and cardiovascular events (6). Cardiovascular effects of ADMA and SDMA in children with CKD are also notable. Brooks et al. (16) observed a strong link between SDMA levels and blood pressure load in adolescents. Chien et al. (17) and Lin et al. (18) associated these markers with arterial stiffness, while Speer et al. (19) suggested that SDMA may cause endothelial dysfunction. Shroff et al. (20) reported increased SDMA content in HDL among pediatric CKD patients, potentially affecting endothelial health. However, their roles as independent cardiovascular risk factors in children remain to be clearly defined. The cardiovascular effects of ADMA occur particularly through its inhibitory effects on endothelial function. ADMA reduces nitric oxide synthesis by acting as an endogenous nitric oxide synthase inhibitor, leading to impaired vascular tone and long-term arterial stiffness (4). This mechanism suggests that elevated ADMA levels in childhood, even before clinical cardiovascular symptoms appear, may reflect subclinical vascular changes. Piechowicz et al. (5) also reported significant associations between ADMA levels and vascular parameters.

This study evaluated serum ADMA and SDMA levels in children with CKD and explored their associations with disease severity and renal function. Our results confirm that both biomarkers are significantly elevated in pediatric CKD compared to healthy controls, with ADMA showing stronger stage-specific increases, especially in stage 4 disease.

While previous studies have reported associations between these dimethylarginines and renal dysfunction, the contribution of our work lies in its relatively large pediatric cohort, the use of UHPLC-ESI-MS/MS as a high-precision method for simultaneous biomarker quantification, and detailed stage-specific *post-hoc* comparisons (21–23). Importantly, the inclusion of a well-matched control group enabled us to demonstrate that ADMA levels become particularly discriminative in advanced stages of CKD.

Our findings are consistent with earlier reports that demonstrated correlations between dimethylarginines and reduced eGFR. However, the stage-specific analysis in our study underlines the stronger role of ADMA as a marker of advanced CKD in children. Although our results largely replicate known associations, they add robust data from a Middle Eastern pediatric population, contributing to the global evidence base.

### Limitations

This study has several limitations. First, although UHPLC-ESI-MS/MS is considered a gold standard method with high specificity and sensitivity, we did not perform independent validation using other techniques such as ELISA, which may limit external reproducibility. Second, we did not assess mechanistic regulators of dimethylarginines, such as PRMT or DDAH enzymes. Such analyses, for example through Western blotting or immunohistochemistry, could have strengthened the biological plausibility of our findings. Third, this was an observational study, and no *in vitro* or *in vivo* experiments were conducted to directly demonstrate whether ADMA and SDMA play functional roles in CKD progression or vascular dysfunction in children. Finally, the relatively small number of stage 4 CKD patients limits generalizability, and biological variability or pre-analytical factors (e.g., fasting status, timing of sample collection) may have influenced biomarker levels.

Despite these limitations, our study provides valuable pediatric evidence confirming that serum ADMA and SDMA levels increase with worsening kidney function. ADMA, in particular, shows strong associations with disease severity, underscoring its potential as a clinically useful marker for monitoring pediatric CKD. These findings contribute to the growing body of literature supporting dimethylarginines as biomarkers for risk stratification and disease progression in children.

In conclusion, this study demonstrates that ADMA and SDMA could serve as valuable biomarkers for diagnosing and monitoring CKD progression in children. Particularly, ADMA is a significant parameter related to endothelial dysfunction and renal progression. Clinical use of these biomarkers could offer new perspectives in diagnosing and following pediatric CKD.

### Teaching points

ADMA and SDMA levels are significantly associated with disease severity and renal dysfunction in pediatric CKD.

ADMA levels increase markedly with advancing CKD stages, suggesting its potential as a biomarker of disease progression.

ADMA and SDMA may be helpful as biochemical markers for the early detection of renal impairment in children.

## Data Availability

The raw data supporting the conclusions of this article will be made available by the authors, without undue reservation.
